# Evaluation of an Epitypified *Ophiocordyceps formosana* (*Cordyceps s.l.*) for Its Pharmacological Potential

**DOI:** 10.1155/2015/189891

**Published:** 2015-09-15

**Authors:** Yen-Wen Wang, Tzu-Wen Hong, Yu-Ling Tai, Ying-Jing Wang, Sheng-Hong Tsai, Pham Thi Kim Lien, Tzu-Ho Chou, Jui-Ya Lai, Richard Chu, Shih-Torng Ding, Kenji Irie, Tsai-Kun Li, Shean-Shong Tzean, Tang-Long Shen

**Affiliations:** ^1^Department of Plant Pathology and Microbiology, National Taiwan University, Taipei 10617, Taiwan; ^2^Mucho Biotechnology Inc., Taipei 10684, Taiwan; ^3^Graduate School of Comprehensive Human Sciences, University of Tsukuba, 1-1-1 Tennodai, Tsukuba 305-8575, Japan; ^4^Department of Animal Science and Technology, National Taiwan University, Taipei 10672, Taiwan; ^5^Center for Biotechnology, National Taiwan University, Taipei 10617, Taiwan; ^6^Laboratory of Molecular Cell Biology, Faculty of Medicine, University of Tsukuba, 1-1-1 Tennodai, Tsukuba 305-8575, Japan; ^7^Graduate Institute of Microbiology, College of Medicine, National Taiwan University, Taipei 10051, Taiwan

## Abstract

The substantial merit of *Cordyceps s.l.* spp. in terms of medicinal benefits is largely appreciated. Nevertheless, only few studies have characterized and examined the clinical complications of the use of health tonics containing these species. Here, we epitypified *C. formosana* isolates that were collected and characterized as *Ophiocordyceps formosana* based on morphological characteristics, molecular phylogenetic analyses, and metabolite profiling. Thus, we renamed and transferred *C. formosana* to the new protologue *Ophiocordyceps formosana *(Kobayasi & Shimizu) Wang, Tsai, Tzean & Shen *comb. nov.* Additionally, the pharmacological potential of *O. formosana* was evaluated based on the hot-water extract from its mycelium. The relative amounts of the known bioactive ingredients that are unique to *Cordyceps s.l.* species in *O. formosana* were found to be similar to the amounts in *O. sinensis* and *C. militaris*, indicating the potential applicability of *O. formosana* for pharmacological uses. Additionally, we found that *O. formosana* exhibited antioxidation activities *in vitro* and *in vivo* that were similar to those of *O. sinensis* and *C. militaris*. Furthermore, *O. formosana* also displayed conspicuously effective antitumor activity compared with the tested *Cordyceps s.l.* species. Intrinsically, *O. formosana* exhibited less toxicity than the other *Cordyceps* species. Together, our data suggest that the metabolites of *O. formosana* may play active roles in complementary medicine.

## 1. Introduction

The entomopathogenic fungus* Cordyceps s.l.* (*sensu lato*) species, including* Ophiocordyceps sinensis* and* Cordyceps militaris*, are among the prestigious traditional Chinese medicines and have a long history of use as tonics and folk medicines that can be applied to cancer and diabetes treatments and used as antioxidants among other uses [1]. Rather than the safety of the use of* Cordyceps s.l.* (*sensu lato*) species, much attention has been focused on their high value due to their pharmacological and therapeutic benefits in the treatment of respiratory and cerebrovascular diseases [2], enhancement of immunoregulatory function [3], regulation of liver metabolism [4], and treatment of cancer [5]. In this regard, in recent years, much effort has been devoted to discovering the bioactive ingredients, pharmacological efficacies, and mechanisms of action of the secondary metabolites that are derived from* Cordyceps s.l.* spp. For instance,* Cordyceps s.l.* spp. have been well characterized as possessing abundant bioactive compounds, such as cordycepin, D-mannitol (cordycepic acid), adenosine, polysaccharides, vitamins, and enzymes. Among these compounds, cordycepin (3-deoxyadenosine) is uniquely produced in* Cordyceps* spp., is a key active constituent of medicines, and confers broad-spectrum biological activity due to the steroidogenic [6] and antitumor proliferation and antimetastasis activities [7, 8] that are mediated by its antioxidation effects [9]. Additionally, reactive oxygen species (ROS) have been found to contribute to cellular necrosis and a variety of pathological conditions, including cancer [10], degenerative diseases in neurons [11], hepatopathies [12], atherosclerosis [13], and even aging [14]. Therefore,* Cordyceps s.l.* species have been repeatedly reported as antioxidants [15] due to their free radical scavenging abilities.

*Cordyceps s.l.* is a large paraphyletic group in Clavicipitaceae* s.l.* that comprises species with a wide range of hosts that span from insects and spiders to* Ophiobolus* and truffle-like fungi [16, 17]. Initially, the classification was based on the color and shape of the stromata, the perithecia, the sizes and shapes of asci and ascospores, and the host species. According to these characteristics, Kobayasi classified the genus into three subgenera,* Cordyceps*,* Ophiocordyceps,* and* Neocordyceps* [17]. Later, two extra subgenera were introduced,* Racemella* and* Cryptocordyceps*, by Mains [18]. Since then, the definitions of several original subgenera have experienced drastic changes. Specifically, for rapid and accurate identification and phylogenetic studies, molecular markers, such as internal transcribed spacers (ITSs), RNA polymerase II (RPB), and elongation factor (EF), have been widely used [19, 20]. In 2007, Sung et al. revised the classification based on phylogenetic analyses of multiple genes. Consequently, Clavicipitaceae* s.l.* was divided into three families, Cordycipitaceae, Clavicipitaceae* s.s.*, and Ophiocordycipitaceae. Among these families,* Cordyceps s.l.* was separated into several genera that included* Cordyceps s.s.*,* Elaphocordyceps*,* Metacordyceps,* and* Ophiocordyceps*. Indeed, the connections between the morphological characteristics and the molecular phylogeny provide invaluable information about* Cordyceps s.l.* that can be used to identify species and genera based on molecular evidence [21].

*Ophiocordyceps formosana* was first reported in 1981 by Kobayasi [22], and the etymology referred to its original discovery as an indigenous* Cordyceps s.l.* fungus in Taiwan. However, the characteristics of* O. formosana* were poorly documented with respect to the morphological description. Physiological and biochemical analyses and descriptions of its pharmacological applications were completely absent. Accordingly, the growth habits and morphological features of the known herbal* Cordyceps s.l.* species attracted our attention for their potential medicinal applications. Nevertheless, until now, only few and scattered findings regarding* O. formosana* have been reported in Taiwan and China, and sufficient specimens and samples for further characterization and investigation are lacking.

The safety and medicinal efficacy of* Cordyceps s.l.* and related products have been the focus of the development of complementary and traditional medicine. However, due to its limited distribution, high price, overexploitation, and difficulty related to artificial culture,* O. sinensis* has become an endangered species. Here, we further established an epitypified* O. formosana* that is phylogenetically related to* O. sinensis*. Additionally, we evaluated its effective pharmacological potentials in terms of antitumor and antioxidation bioactivities to elucidate the medicinal merit of* O. formosana* as a substitute for* O. sinensis* and* C. militaris*.

## 2. Materials and Methods

### 2.1. Sample Collection and Maintenance

*Ophiocordyceps formosana* specimens were collected from mummified darkling beetle bodies residing in decayed wood in Lala Shan, Taoyuan County, Taiwan (latitude: 24°42′1.20′′N, longitude: 121°25′49.20′′E) in the summer of 2013. The collected samples were immediately photographed and brought back to the Applied Mycology Laboratory of the National Taiwan University in Taipei for spore and mycelium isolation according to a standard decontamination procedure. Several isolates, including* O. formosana* MUCHO 815 and* O. formosana* NTU00035, were successfully obtained and cultivated on PDA and S-DAY plates at 25°C. Every 3-4 weeks, the colonies were harvested. The harvested colonies were first desiccated at 55°C overnight and then stored in a desiccator for future use. To maintain and preserve the* O*.* formosana* samples, small cubic mycelia-containing agars were routinely transferred to new PDA or S-DAY plates for maintenance.* Cordyceps militaris* mycelia and fruiting bodies were obtained from Mucho Biotechnology Inc. as described previously [23]. The mycelia and fruiting bodies of* O. sinensis* were purchased from Tongrentang Co., Ltd., Beijing, China.

### 2.2. Micromorphological Characteristic Examination

Microscopic examinations were performed via cryosectioning and examination under a compound light microscope (Olympus, Japan). The samples were fixed with 4% formaldehyde in PBS for one day with one change of solution. After the samples were mounted in optimal cutting temperature (OCT) compound, cryosectioning was performed with a LEICA CM3050-S cryostat (Leica Biosystems, Germany) to obtain sections ranging in thickness from 5 *μ*m to 10 *μ*m. The sections were examined and photographed under an Olympus BH2 light microscope (Olympus, Japan) equipped with a Canon-ds126 camera.

### 2.3. Genomic DNA Extraction from* Cordyceps* spp

The genomic DNA extraction from* Cordyceps* spp. was performed according to a modified version of the protocol of Doyle [24]. Briefly, either the fungal samples were directly cut down from the stroma and stalks of* Cordyceps* or mycelia were harvested from culture plates. The collected samples were lyophilized and ground to powder with a pestle with 500 *μ*L of hot CTAB buffer (2% CTAB, 1.4 M NaCl, 20 mM EDTA, 100 mM Tris at pH 8.0, and 2% PVP-40) in combination with 3 *μ*L of fresh *β*-mercaptoethanol. After incubation at 60°C for 20 minutes, 500 *μ*L of chloroform : isoamyl alcohol (CI; 24 : 1) was added and mixed gently for 10 minutes prior to centrifugation at 13,200 g for 2 minutes. The supernatant was carefully transferred to a new Eppendorf tube following the administration of a 0.6x volume of isopropanol. Next, the mixture was allowed to incubate at −20°C for 30 minutes to precipitate the DNA. The DNA was harvested by centrifugation at 13,200 g for 30 minutes along with a single 75% cold ethanol wash, drying with a vacuum (LABCONCO, USA), and resuspension in 50 *μ*L of ddH_2_O.

### 2.4. DNA Amplification and Sequencing

PCR was employed for the amplification of specific DNA fragments as described below. In general, the PCR reaction mixtures contained 2.5 *μ*L of 10x reaction buffer, 1 *μ*L of 10 *μ*M of each specific primer (see Supporting Table 1 in Supplementary Material available online at http://dx.doi.org/10.1155/2015/189891), 0.5 *μ*L of 10 mM dNTPs, 2 *μ*L of template DNA, and 0.3 *μ*L of 1 U Taq polymerase in a total volume of 25 *μ*L. The* RPB1* sequences were amplified using the forward primer cRPB1 and the reverse primer RPB1c-r [25] according to the following steps: denaturation at 95°C for 5 minutes; 40 cycles of 95°C for 1 minute, 55°C for 1 minute, and 72°C for 1 minute; and an additional extension step at 72°C for 10 minutes. The* RPB2* sequences were amplified with the forward primer fRPB2-5f and the reverse primer fRPB2-7cr [26] according to the following steps: denaturation at 95°C for 5 minutes; 40 cycles of 95°C for 50 seconds, 55°C for 1 minute, and 72°C for 90 seconds; and an additional extension step at 72°C for 10 minutes. The* EF1-α* sequences were amplified using the forward primer EF1-2218R and the reverse primer EF1-983F [27] according to the following steps: denaturation at 94°C for 5 minutes; 40 cycles of 94°C for 1 minute, 46°C for 30 seconds, and 72°C for 2 minutes; and an additional extension step at 72°C for 7 minutes. The PCR products were electrophoresed and eluted for sequencing using an ABI 3730XL system (Applied Biosystems, MA, USA). The obtained sequences were then assembled and trimmed using a ContigExpress of vector NTI.

### 2.5. Phylogenetic Analysis

The nucleotide sequences of three genes, that is, the largest and second largest subunits of RNA polymerase II (*RPB1*,* RPB2*) and translation elongation factor 1*α* (*EF1-α*), were either obtained from our sequenced PCR products of the* O. formosana* isolates, retrieved from the NCBI database following the criteria described by Sung et al. [21], or acquired by whole genome ortholog BlastNs of* C. militaris* CM01. A total of 51 species/isolates were included in this study (Supporting Table 3). The nucleotide sequences of these three genes were concatenated, aligned, and tested in models using MEGA 6.0 [28]. The best model according to the AIC and BIC values was the GTR+I+G model. The aligned sequences were analyzed using CIPRES portal-based Raxml blackbox (RAxML 7.2.7) [29] for maximum likelihood (ML) tree searching with the default settings, an estimation of the gamma distribution, invariable sites, and Bayesian inference of the phylogeny with MrBayes 3.2.3 with the parameters obtained from the model test. The resulting phylogenetic trees were saved and plotted using Adobe Illustrator CS3.

### 2.6. *Cordyceps* Extract Preparation and HPLC Analysis

All of the desiccated* Cordyceps* samples that were subjected to HPLC (HITACHI, D-2000 HSM, Japan) analyses for* de novo* secondary metabolites were pulverized with a homogenizer at 1,300 rpm and subsequently stored in a humidity-controlled desiccator at room temperature. The preparation of the* Cordyceps* extracts was performed according to the standard operation procedure described below. Briefly, 1 g of* Cordyceps* powder was dissolved with deionized water at a solid-to-solvent ratio of 1 : 40 by vortexing for 30 seconds in a 50 mL falcon tube (BD Biosciences, USA). Next, the extraction was performed with 50°C hot water reflux for 2 hours (including the first 30 minutes of ultrasonication). After 20 min of centrifugation at 4,000 rpm (approximately 3,200 g), the supernatant was collected and filtered with a 0.22 *μ*m filter. The resulting extracts were directly subjected to HPLC analyses or stored at −20°C.

High-performance liquid chromatography (HPLC) was performed on RP-18 column (150252 Purospher STAR RP-18 endcapped (5 *μ*m) LiChroCART 250-4, Merck) at a flow rate of 1 mL/min. The composition of the mobile phase was 20% methanol/H_2_O. Three of the indicated components, D-mannitol, adenosine, and cordycepin, were detected at 260 nm using a DAD detector and the corresponding retention times of the individual standard compounds in the same HPLC conditions. Quantitation was preformed according to the established standard curve of each compound.

### 2.7. Cell Culture and MTT Assay

The MTT assay was used to measure the abilities of the* Cordyceps* extracts to inhibit human cancer cell proliferation. Approximately 5 × 10^3^ cells were seeded onto each well of a 96-well plate containing 200 *μ*L of culture medium (per well) 24 hours before treatment. After exposure to various concentrations of the indicated extracts for 96 hours, the cells were incubated with 20 *μ*L of MTT (Thiazolyl Blue Tetrazolium Bromide, Sigma, USA) solution for 4 hours. Cell proliferation was measured by the absorption at 570 nm using a spectrophotometer (SpectraMax 190 UV-Vis Microplate, Molecular Devices, USA). The measurements were performed in triplicate. The percentages of viable cells were calculated as (sample OD_570_ nm/Mock OD_570_ nm) × 100 (%) [30].

### 2.8. DPPH Antioxidation Assay

The scavenging of DPPH radicals was estimated by vigorously mixing equal amounts of the* Cordyceps* extract and DPPH solution (0.1 mM in methanol). The mixture was then incubated at room temperature for 50 minutes before measurement at 517 nm using a spectrophotometer (SpectraMax 190 UV-Vis Microplate, Molecular Devices, USA). The scavenging activity was determined by comparing the blank (100%) that contained only DPPH and solvent. The scavenging efficiency percentages were calculated as (sample OD_517_ nm/Mock OD_517_ nm) × 100 (%) [31].

### 2.9. Cell-Based ROS Scavenging Assay

Approximately 5 × 10^4^ CHO-K1 cells were seeded in each well of a 12-well plate and exposed to the indicated* Cordyceps* extracts for 1 hour after 16 hours of serum starvation. Here, the 10 mM N-acetylcysteine treatment served as a positive control. After H_2_O_2_ (1800 *μ*M) challenge for 5 minutes, the cells were incubated with fluorescent probes (CM-H2DCFDA, Sigma, USA) for 30 minutes in a 37°C incubator (light was excluded). Finally, the cells were trypsinized and subjected to ROS quantification via the detection of the DCF levels, which corresponded to the relative ROS amounts at 525 nm using a flow cytometer (BD FACSCanto, USA).

### 2.10. Tumor Xenografts

An* in vivo* tumorigenesis assay was conducted as previously described [32]. Briefly, 5 × 10^6^ MDA-MB-231 breast cancer cells were inoculated into the left thighs of 10-week-old nude mice (BALB/cAnN.Cg-*Foxn1* ^*nu*^/CrlNarl) via subcutaneous injections. The oral gavage treatments with* O. formosana* extract began daily immediately after 10 days when the tumor growth was palpable. The mice were divided into three groups: two groups were treated with* O. formosana* water extracts at concentrations of 1x (25 mg/mL, 100 *μ*L/day) and 5x (125 mg/mL, 100 *μ*L/day), and a control group was treated with PBS. The tumor sizes were measured every four days and calculated with the formula tumor size = 0.4 × length × width^2^ according to previous reports [33]. Statistical strategy was based on Ku's study [34]. After 26 days of* O. formosana* water extract treatment, the tumors in the mice were photographed.

### 2.11. Statistical Analyses

All data are expressed as the means ± the SEMs based on at least three independent experiments. The statistical analyses were performed using one-way analyses of variance (ANOVA) followed by Tukey's tests, and significance was measured as indicated.

## 3. Results

### 3.1. Collection and Morphological Features of* Ophiocordyceps formosana*

**Figure 1:**
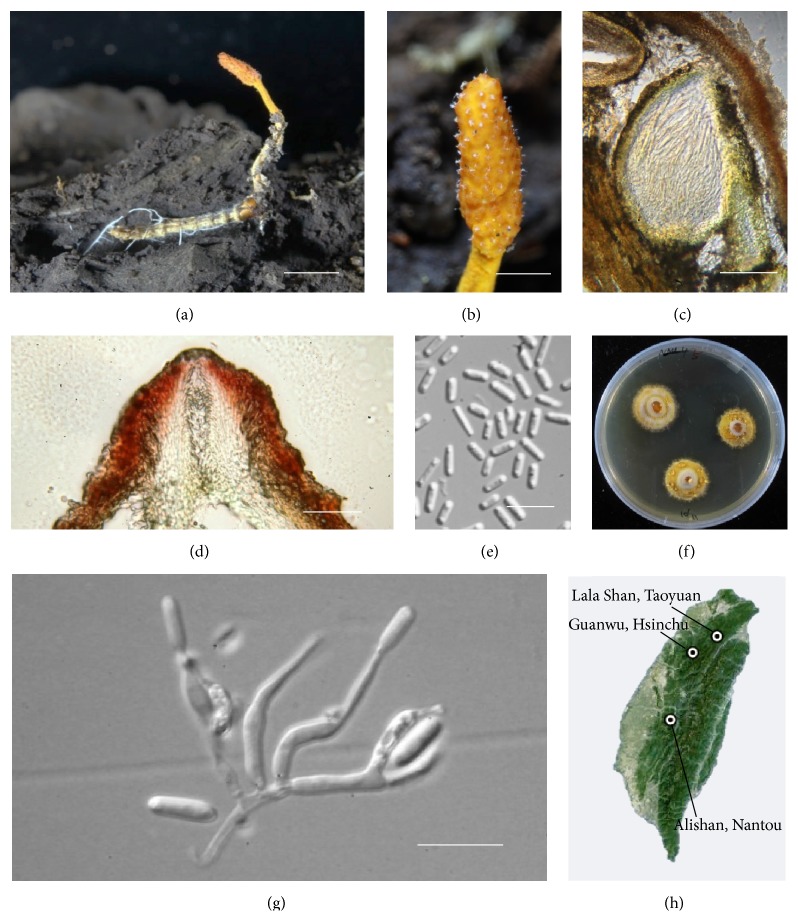
Morphological and anatomical features of the* Ophiocordyceps formosana* collected from Taoyuan County, Taiwan. (a) The fungal ascostromata arise from the host Tenebrionoidea insect corpses with cylindrical stalks. Bar = 10 mm. (b) The orange stromatal head exhibits an oblong shape. Bar = 3 mm. (c) An ovoid perithecium embedded in a stroma is shown in cross section. The asci reside within the ovoid perithecium and obliquely align with the perithecium. Bar = 100 *μ*m. (d) The ostiole of a perithecium showing a narrow opening in the neck surrounded by a thick perithecial wall. Bar = 25 *μ*m. (e) Cylindrical partspores derived from fragmented ascospores. Bar = 10 *μ*m. (f) The colonies were velutinous or floccose with exudating droplets and orange to white in color on a PDA culture plate. (g) A* Hirsutella* anamorph of* O. formosana*; the branched conidiophore is curl-shaped and bears baciloid-shaped conidia. Bar = 10 *μ*m. (h) The documented habitats of* O. formosana* specimens in Taiwan [22, 35] (source: Taiwan; 23°46′50.90′′N and 120°57′50.54′′E from Google Earth).

We collected* Cordyceps* spp. from varied potential niches in Taiwan to assess their potential medicinal values and applications. In Taoyuan County of Taiwan (GPS: altitude 1600 m, latitude: 24°42′1.20′′N, longitude: 121°25′49.20′′E; collectors: Y.-W. Wang, S.-H. Tsai, T.-W. Hong, J.-Y. Lai, T.-L. Shen, and others; Figure 1(h)), two* Ophiocordyceps formosana* samples were found growing on darkling beetle larvae (Tenebrionoidea) that resided on fallen decayed wood in August of 2013. Based on examination of the macroscopic and microscopic details of the newly collected* O. formosana*, the morphological features were described and documented as shown in Figure 1.

Obviously, the ascostromata arose from the heads or abdomens of the infected insect hosts (here, darkling beetle larva of the Tenebrionoidea superfamily), and the stalks exhibited long-cylindrical shapes of 10–30 mm × 0.5–2 mm in size that were orange (6A7) in color and bore short hairs but lacked membranous sheaths. The oblong ascostromata heads were 4–6 mm × 1–4 mm in size and largely consisted of pseudoparenchymatous epidermal tissues. Moreover, the perithecia were embedded within the stromata and were brownish orange (7C8) and ovoid shaped (360–480 × 240–320 *μ*m) with short necks, with ostioles of 60 *μ*m in width. The perithecial wall was 20 *μ*m thick underneath with approximately 40 *μ*m-thick epidermal tissue. Furthermore, the asci displayed long-cylindrical shapes with attenuated bases (6.5–7.9 *μ*m × 160–240 *μ*m) and thick dome-shaped apices (3.9–5.3 × 3.2–4.6 *μ*m) along with narrow slits. The asci contained eight ascospores and hyaline, were cylindrical and filamentous, and were often fragmented into more than 10–20 cylindrical and truncated partspores (2.6–3.0 × 6.5–7.3 *μ*m) at maturity. Colonies grown on potato dextrose agar (PDA) were orange (5A7) to white in color and pulvinate to umbonate in shape and exhibited velutinous to floccose mycelia that were whitish light-orange to pink in color and exudation droplets. The anamorphic state was similar to* Hirsutella* type, and the conidiogenous cells were monophialidic, hyaline, and elongated ampuliform-shaped (1.5–2.3 × 8.6–17.0 *μ*m). The conidia were hyaline and cylindrical (1.6–2.3 × 3.6–6.9 *μ*m in size).

### 3.2. Molecular Identification of* Ophiocordyceps formosana* by* RPB1*,* RPB2*, and* EF1-α*

In addition to the morphological features, we amplified the 790-, 1062-, and 1068-base pair (bp) nucleotide sequences of the* RPB1*,* RPB2*, and* EF1-α* genes from the isolates of* O. formosana*, respectively, by polymerase chain reaction (PCR) with the corresponding specific primers listed in Supporting Table 1. The obtained and sequenced genes were subjected to BLAST in the NCBI database (http://www.ncbi.nlm.nih.gov/). The top hits of the 3 sequences were* Cordyceps formosana* TNM F13893,* Ophiocordyceps coenomyia* NBRC 106964, and* Cordyceps formosana* TNM F13893 with identities of 100% (635/635), 90% (956/1065), and 99% (847/849), respectively. Although the top hit for RPB2 was not* Cordyceps formosana*, the sequence shared 100% identity with the* Cordyceps formosana* TNM F13893 with 47% coverage (Supporting Table 2). The ITS sequences were not utilized due to time and reagent limitations. However, the multigene analysis results of the three genes were sufficient to indicate that our collected specimens were identical to* Cordyceps formosana* TNM F13893 as verified by the molecular markers.

**Figure 2:**
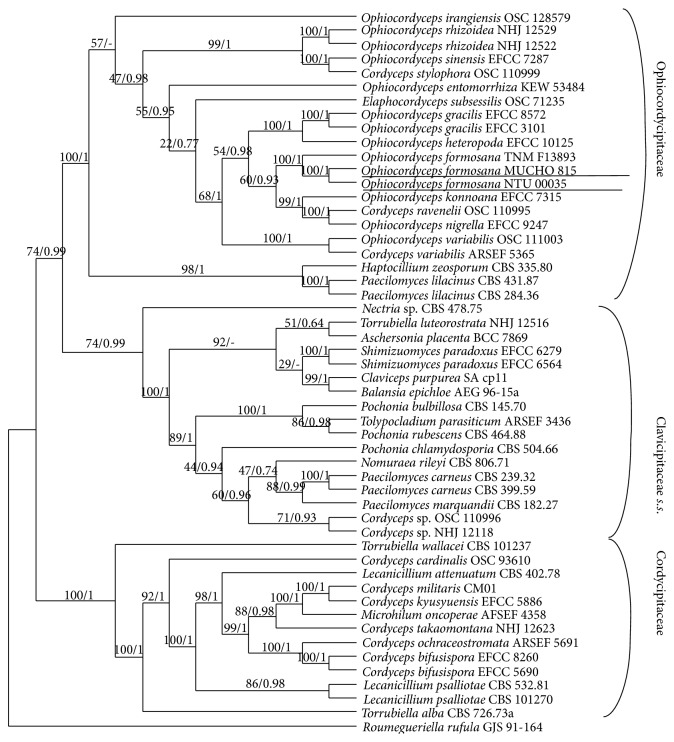
Phylogenetic tree of* Cordyceps s.l.* Phylogenetic cladogram of* Cordyceps s.l.* showing the three newly created families, Clavicipitaceae* s.s.*, Cordycipitaceae, and Ophiocordycipitaceae, as inferred by the maximum likelihood (ML) method and Bayesian inference of the phylogeny using three concatenated genes (*RPB1*,* RPB2*, and* EF1-α*). The bootstrap support values are denoted on the branches in front of the posterior probabilities. The underlined* Ophiocordyceps formosana* NTU 00035 and MUCHO 815 samples were collected from Taiwan in this study.

Indeed, the phylogenetic trees based on the maximum likelihood method and Bayesian principles resulted in two identical trees with different support strengths. A maximum likelihood bootstrap tree is shown in Figure 2, and the posterior probability ML bootstrap support values are denoted after the slashes. The topologies of* Cordyceps s.l.* in the three newly created families, Ophiocordycipitaceae, Cordycipitaceae, and Clavicipitaceae* s.s*., were largely congruent with the results of Sung et al. [21].* O. formosana* resided within the Ophiocordycipitaceae clade with a strong bootstrap support value (BS = 10/PP = 1) rather than in the other allied taxa of Cordycipitaceae and Clavicipitaceae* s.s*. Phylogenetically,* O. formosana* obviously exhibited a closer relatedness to* O. sinensis* than to* C. militaris*. However, in contrast,* C. militaris* was placed in the Cordycipitaceae clade, which was distantly related to* O. sinensis* and* O. formosana*. Intriguingly, the* O. formosana* collected in Taiwan from the varied geographic regions in this study were in the clade of* O. formosana* isolated by another group. Based on the unambiguous morphological traits and molecular phylogenetic study, we recombined the previously erected* Cordyceps formosana* as* Ophiocordyceps formosana*, and the epitypic specimen was designated as* O. formosana* MUCHO 815.

### 3.3. Chemical Profiles of the* Ophiocordyceps formosana *Water Extracts

**Figure 3:**
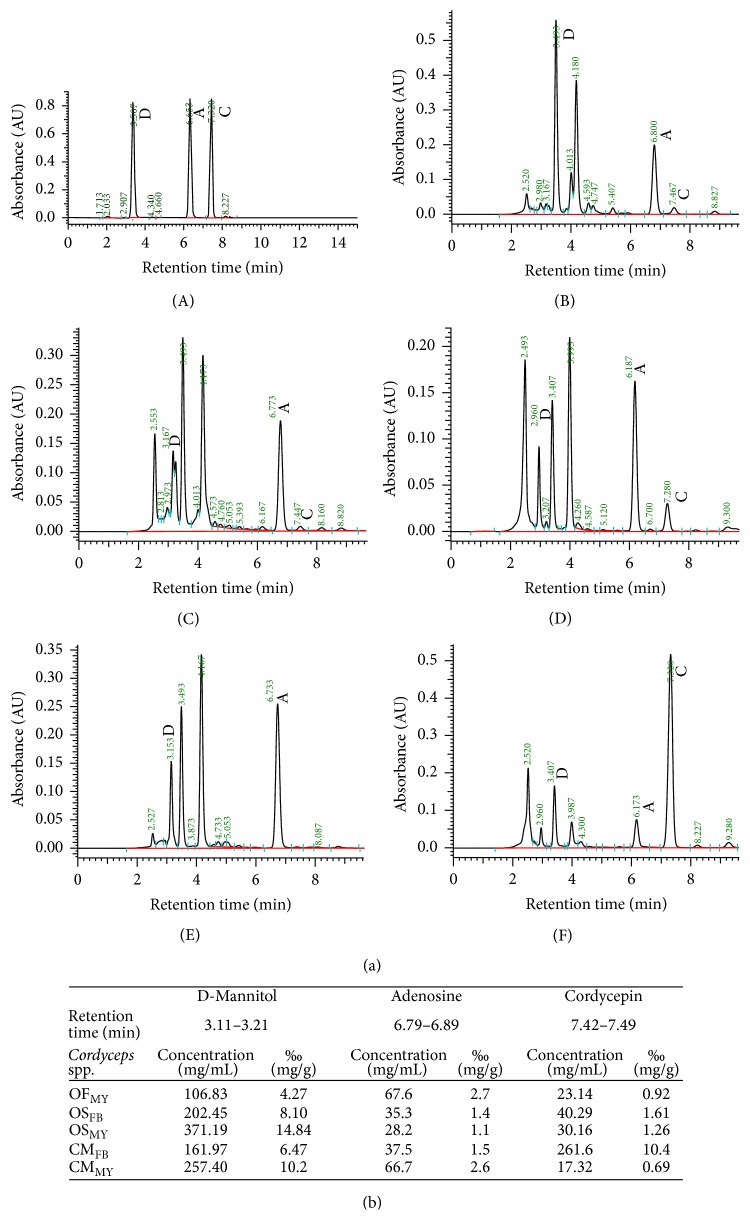
HPLC profiles and analyses of extracts from various* Cordyceps* species. (a) The HPLC retention times are shown for D-mannitol (designated as “D”), adenosine (designated as “A”), and cordycepin (designated as “C”) (A). The HPLC profiles extracted from the* O. formosana* mycelia (B, OF_MY_), the* O. sinensis* mycelis (C, OS_MY_) and fruiting bodies (D, OS_FB_), and the* C. militaris* mycelia (E, CM_MY_) and fruiting bodies (F, CM_FB_). (b) The quantitative results for the indicated compounds were calculated according to the area under the peak that corresponded to the standard calibration;* r* ^2^ > 0.999.

The chemical profiles of the extracts from the mycelia and fruiting bodies of the various* Cordyceps* species, including* O. sinensis* and* C. militaris*, were compared with* O. formosana*. To perform the investigations, the hot water extracts of the tested* Cordyceps* samples were subjected to HPLC analyses. The HPLC profile of* O. formosana* MUCHO 815 (Figure 3(a)) was reminiscent of those of* O. sinensis* and* C. militaris*, which indicated that the biochemical properties of* O. formosana* were similar to those of other well-known* Cordyceps* species. Additionally, we compared three predominate metabolites that were unique in* Cordyceps* spp., cordycepin, adenosine, and D-mannitol. As shown in Figure 3(b),* O. formosana* MUCHO 815 possesses amounts of these three major bioactive compounds in terms of quantities and percentiles that were similar to other* Cordyceps* species. Collectively, we epitypified* O. formosana* based on detailed studies of its morphological, molecular, and biochemical features.

### 3.4. The* Ophiocordyceps formosana* Water Extract Displayed Antioxidation Activity

**Figure 4:**
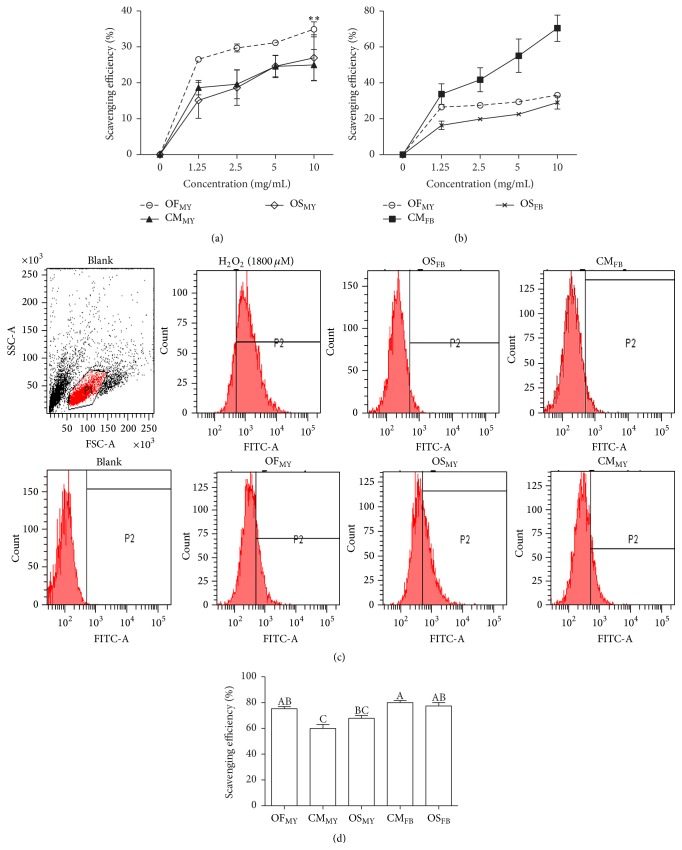
The* in vitro* and* in vivo* antioxidation activities of the extracts from various* Cordyceps* species. (a) The* in vitro* DPPH antioxidant activities of the water extracts of* O. formosana*,* C. militaris*,* and O. sinensis* mycelia (designated as OF_MY_, CM_MY_, and OS_MY_, resp.). (b) The DPPH antioxidant activity of the* O. formosana* mycelial water extract compared to those of the water extracts of the* C. militaris* and* O. sinensis* fruiting bodies (designated as CM_FB_ and OS_FB_, resp.). (c, d) The* in vivo* cell-based ROS scavenging effect as measured by flow cytometry (c) and the quantification results (d). ^ABC^Bar graphs without the same letter are significantly different from one another in each group (*p* < 0.05).

*Ophiocordyceps formosana* displayed biochemical features that were analogous to those of other valuable medicinal* Cordyceps *spp.* s.l.* We furthermore examined its biological and pharmacological properties. The nature of the antioxidant activity of the hot water extract from* O. formosana* was evaluated based on DPPH assay and cell-based ROS scavenging bioactivity. As shown in Figures 4(a) and 4(b), the hot water extract of* O. formosana* MUCHO 815 apparently exhibited a strong antioxidant capability compared with the extracts from the mycelia of* O. sinensis* and* C. militaris* (Figure 4(a)) that was even comparable with that of the extract derived from the* O. sinensis* fruiting body (Figure 4(b)). Coincidently, an* in vivo* cell-based ROS scavenging assay using CHO-K1 cells revealed that* O. formosana* exhibited a nonstatistically significantly greater antioxidant effect than the mycelial extracts (Figure 4(c)). Notably, the extract from the* O. formosana* mycelia was also comparable to the extracts derived from the* O. sinensis* and* C. militaris* fruiting bodies (Figure 4(d)). Our data imply that* O. formosana* may serve as an alternative and/or substitute for tonic and medicinal uses of* O. sinensis* and* C. militaris*.

### 3.5. The* Ophiocordyceps formosana *Water Extract Exhibits Anticancer Activity

**Figure 5:**
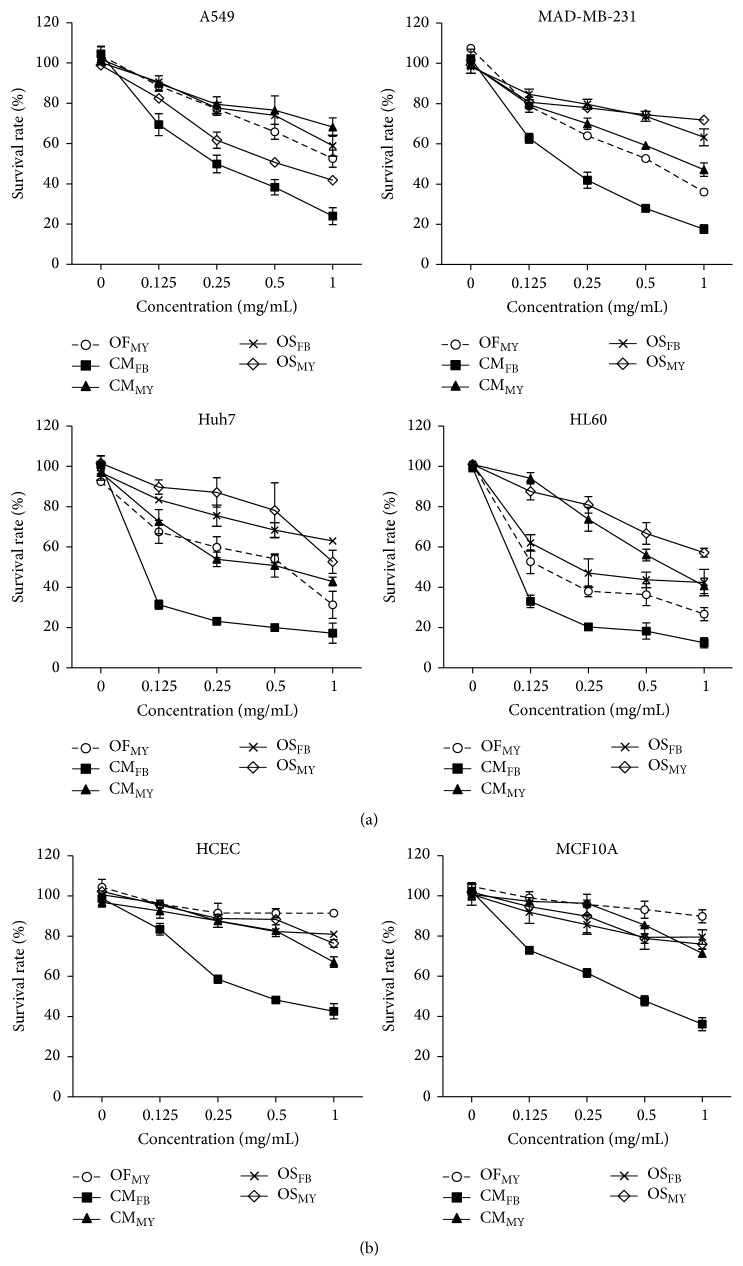
Antitumor activities of the extracts from various* Cordyceps* species. (a) Survival rates of various cancer cell lines, including the human lung cancer A549, human breast cancer MDA-MB-231, human liver cancer Huh7, and human leukemia HL60 cell lines exposed to the water extracts of* O. formosana *(OF_MY_),* C. militaris* (CM_MY_ and CM_FB_), or* O. sinensis *(OS_MY_ and OS_FB_). (b) The survival rates of human normal cell lines, human corneal epithelial HCEC cells, and human lung epithelial MCF10A cells that were exposed to the water extracts of* O. formosana*,* C. militaris,* or* O. sinensis*.

To determine the anticancer potential of* O. formosana*, the hot water extracts from the mycelia and fruiting bodies of* O. formosana*,* O. sinensis,* and* C. militaris* were tested against various human cancer cell lines, including A549 lung cancer, MDA-MB-231 breast cancer, Huh7 liver cancer, and HL-60 leukemia cells, at different dosages. As shown in Figure 5(a), the* O. formosana* extract exerted anticancer activities IC_50_s of 1.0 mg/mL for the A549 cells, 0.53 mg/mL for the MDA-MB-231 cells, 0.44 mg/mL for the Huh7 cells, and 0.19 mg/mL for the HL-60 cells. Apparently, the* O. formosana* extract exhibited more effective cancer cell suppression activities than the extracts from the* O. sinensis* and* C. militaris* mycelia that were even comparable to those of the extract derived from the* O. sinensis* fruiting bodies in all tested cancer cell lines (Table 1). Strikingly, the* O. formosana* extract yielded the lowest toxicities (maintaining a survival rate greater than 90%) to the tested normal cell lines, which included HCEC normal human corneal epithelial cells and MCF10A normal human breast epithelial cells, among all of the other* Cordyceps *extracts as shown in Figure 5(b).

### 3.6. *In Vivo* Anticancer Activity of the* Ophiocordyceps formosana* Water Extracts

**Figure 6:**
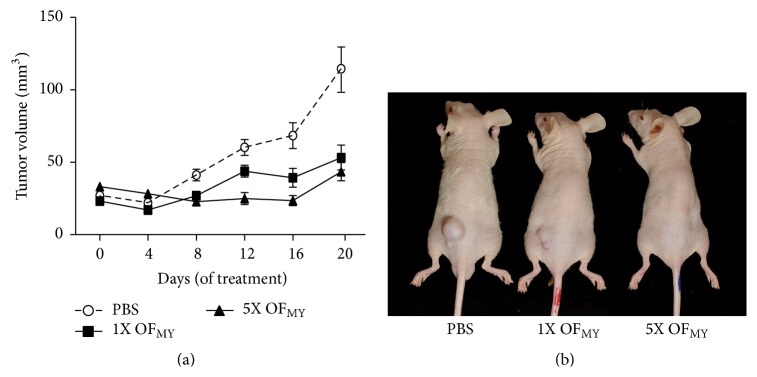
*In vivo* antitumor activities of the extracts from* O. formosana*. (a) Mice were xenografted with human MDA-MB231 breast cancer cells and then orally treated with the water extracts of* O. formosana* (OF_MY_) with 1x (2.5 mg, *n* = 3), 5x (12.5 mg, *n* = 3), or PBS only daily. The tumor sizes were recorded every four days and showed decreases in tumor volumes in accordance with the dosage of treated OF_MY_ water extracts. Note that bar is shown as SEM. (b) Photos of tumors in the mice with or without treatments on day 20.

To further validate the antitumor activities of the extracts from* O. formosana*, we conducted an* in vivo* antitumorigenesis assay using tumor xenograft mouse models. Briefly, nude mice were first implemented with the human MDA-MB-231 breast cancer cells and then treated with the water extracts of* O. formosana* at low (2.5 mg/day) and high doses (12.5 mg/day) daily by oral gavages (Figure 6). The results showed that the treated groups exhibited decreases in tumor size compared to the nontreated group in a dose-dependent manner. Our study highlights the potential future applications of* O. formosana* in tonics and medicines.

## 4. Discussion

*Ophiocordyceps formosana* was originally collected from Xitao, Nantou County, Taiwan, and described and named* Cordyceps formosana* in the subgenus* Eucordyceps* by Kobayasi [17, 22]. Nevertheless,* Ophiocordyceps formosana* did not receive much attention until a revisit based on a new collection found in the Guanwu National Forest Recreation Area of Hsinchu County, Taiwan [35]. Consistent with our discovery, the indigenous* Ophiocordyceps formosana* seemed to prefer to inhabit Tenebrionoidea larva in areas of temperate broadleaf forests at approximately 1500–1800 m in altitude (Figure 1(h)). More recently,* O. formosana* was also found in Huangshan, Anhui Providence of China, by Li et al., which extended the distribution of* O. formosana* beyond Taiwan [36].

Morphologically, our collected samples were nearly almost identical to the holotype of* Cordyceps formosana* described by Kobayasi [22] and Li et al. [36], which therefore justified the identification of the entomopathogen discovered in this study as* C. formosana* (Figure 1). However, the morphology and the genotype of* C. formosana* had not been clearly defined and linked. To address this issue, the nucleotide sequences of the* RPB1*,* RPB2*, and* EF1-α* housekeeping genes that were obtained and sequenced from our fungal samples were aligned and compared with the documented allied sequences of the reported* C. formosana* TNM F13893 to further validate the identities of both fungal specimens. The reason that we used* RPB1*,* RPB2,* and* EF1-α* to infer the species phylogeny was based on a hypothesis that essential genes are more likely to be subjected to the same selection forces, and convergent evolution in amino acid sequences is less likely to occur; this hypothesis has also been supported in several previous studies of the phylogenies of the* Cordyceps* relatives [20, 21]. Other approaches involving the use of a neutral locus, such ITS, should be appropriate for inferring species phylogeny; however, ITS sequences change too fast and are therefore unsuitable for inferring the phylogenies of distantly related species. Indeed, we failed to obtain the ITS sequence of* C. formosana* using the “so-called” conserved primer sequences. Therefore, in this study, we were dealing with distantly related species, so these three genes were selected to infer the species phylogeny. Interestingly, the phylogenetic tree-based maximum likelihood and Bayesian inference phylogenetic methods both revealed the same topology, although, due to restrictions in computer capacity, we were unable to rule out the possibility that the trees were trapped in local optima. However, we replicated two runs of the BI analyses, and the results were identical, which supports our hypothesis that* Ophiocordyceps formosana* resides in Ophiocordycipitaceae. Due to the clear evidence from not only the morphological characteristics but also the molecular linkages, we epitypified* C. formosana* to* O. formosana*. Moreover, the anamorphic state of* Hirsutella* type in culture was also consistent with the description from Li et al. [36]. According to the definition of* Ophiocordyceps* proposed by Kobayasi, the anamorphs of this genus consist of four genera:* Hirsutella*,* Hymenostilbe*,* Paraisaria*, and* Syngliocladium* types [22]. The results of the phylogenetic investigation further support our suggestion for combining and transferring* C. formosana* to the new protologue* Ophiocordyceps formosana* (Kobayasi & Shimizu) Wang, Tsai, Tzean & Shen* comb. nov.* (see Supporting Text 1 for details), although the comparisons of several phylogenetically related* Ophiocordyceps* spp. indicated that* O. formosana* apparently displays a brighter yellow color of its stromata that distinguishes it from other species in this clade (Figure 1). For example,* O. ravenelii* exhibits superficially distributed perithecia and dark brown-colored clavate stromata [37]. The stromata of* O. nigrella* are also dark brown and clavate, but the perithecia are embedded in mycelial tissues [38], whereas* O. konnoana* exhibits yellow, clavate stromata and superficial perithecia [39]. However,* O. heteropoda* and* O. gracilis* exhibit embedded perithecia and capitate stromata [40, 41], which indicates that these two species may be comparatively less related to* O. formosana*. Interestingly, the clade that was phylogenetically allied to* O. formosana* and included* O. ravenelii*,* O. nigrella,* and* O. konnoana* appears to exhibit a similar host preference, that is, Coleoptera larvae. In contrast,* O. heteropoda* and* O. gracilis* parasitize Auchenorrhyncha (cicada) nymphs and Lepidopteran larvae, respectively. Indeed, regarding phytopathogens, linkages between host ranges and evolution have been suggested [42]. Although it is currently recognized as belonging to* Cordyceps s.l.*, the strict host specificities of some of the clades, such as* Elaphocordyceps*, may be due to recent host jumping [21, 43] that might have given rise to strong driving forces for an evolutionarily genetic drift. Moreover, this clade also shares morphological features of the stromata; however, this seems to be only a symplesiomorphic character within* Ophiocordyceps* spp. [43].

The adverse effects of oxidative stress on human health have attracted considerable interest. Indeed, the exposure of organisms or cells to excessive amounts of reactive oxygen species (ROS) results in striking homeostatic imbalances [44]. Eventually, high levels of ROS are capable of inducing cellular and DNA damage, triggering cell apoptosis, and contributing to deleterious complications, such as aging, high blood pressure, inflammatory responses, and even cancer. Two* Cordyceps* spp., that is,* O. sinensis* and* C. militaris*, are commonly used to ameliorate conditions associated with aging [45] and several malignant complications, including lung disorders [46], diabetes [47], kidney failure [48], and cancer [23]. It is believed that the antioxidation activities derived from different preparations or fractions of* Cordyceps* are effective in therapeutics [49]. Using the* in vitro* DPPH assay and the cell-based anti-ROS scavenging assay (Figure 4), the extract from the mycelia of* O. formosana* was found to exhibit prominent antioxidative activities that exceeded those of the mycelia of* C. militaris* and* O. sinensis* and were even comparable to those of the fruiting bodies of* O. sinensis*. Hence, our evidence-based results suggest the value of the complementary and alternative medicine* O. formosana *for oxidation-related complications.

In recent years, increasing amounts of evidence have indicated that* Cordyceps* spp. are competent as alternative antitumor medicines and adjuvants for chemo- and radiotherapy in the treatment of various cancers [50]. Notably, the hot water extract from the mycelia of* O. formosana* exhibits superior antitumor activities against various cancer cell lines via induction of cancer apoptosis (unpublished data), including breast, lung, and liver cancers and leukemia, compared to extracts from the mycelia of* O. sinensis* and* C. militaris* (Figure 5(a)). Moreover, the* O. formosana* mycelial extract even exhibited an anticancer efficacy that was comparable to that of the extract from the* O. sinensis* fruiting bodies (Table 1); therefore, benefits are available for many markets because a massive supply is achievable. Intriguingly, the relatively lower toxicity of* O. formosana* to normal cells renders* O. formosana* as more competent as an alternative medicine for the treatment of cancer (Figure 5(b)). In support of this idea, we have observed no apparent deleterious effects on any of the dissected tissues/organs or in the* in vivo* biochemistries of mice that have been orally treated with* O. formosana* for 14 successive days at concentrations of up to 2 g/kg (unpublished data).

## 5. Conclusions

In conclusion, we established an evidence-based alternative indigenous medicinal* Cordyceps s.l*. named* Ophiocordyceps formosana* that exhibits pharmacological potential as an anticancer and antioxidative stress-related complication agent. With the support of the HPLC analyses, the chemical profile indicated that key bioactive ingredients, including D-mannitol, adenosine, and cordycepin, were present in* Ophiocordyceps formosana* at amounts that were comparable or even exceeded those in other known traditional medicinal* Cordyceps* species. In addition to its high level of pharmacological efficacy,* O. formosana* also exhibited lower toxicity than the documented valuable* Cordyceps* species* O. sinensis* and* C. militaris*. Together, these findings support the notion that this epitypified* Ophiocordyceps formosana* (*Cordyceps s.l.*) can serve as an alternative medicinal fungus.

## Supplementary Material

Degenerated primer sequences (Supporting Table 1) derived from the largest subunit of RNA polymerase II (RPB1), the second large subunit of RNA polymerase II (RPB2) and translation elongation factor-1*α* (EF1-*α*) genes were synthesized according to previous reports (30, 31, 32) and used for the molecular identifications of Cordyceps spp, (s. l.), including Ophiocordycpes formosana. The amplified 790-, 1062-, and 1068-base pair (bp) nucleotides, respectively, from our collected samples were sequenced and subjected to BLAST in the NCBI database (). The closest species and strains matched, NCBI accession number, and nucleotide identify of the 3 sequences were listed (Supporting Table 2).
A list of nucleotide sequences of the 3 genes (i.e. RPB1, RPB2, and EF-1a) from 51 species/isolates was retrieved from the NCBI database following the criteria described by Sung et al or obtained from our sequenced PCR products of the *O. formosana* isolates (Supporting Table 3).

## Figures and Tables

**Table 1:** The IC_50_ values for each of the extracts of the various *Cordyceps* spp. with antitumor activities.

Cell line (IC_50_)	OF_MY_	CM_FB_	CM_MY_	OS_FB_	OS_MY_
A549	1.00 ± 1.09	0.28 ± 0.06	1.70 ± 0.21	1.31 ± 0.62	0.54 ± 0.57
MDA-MB-231	0.53 ± 0.15	0.20 ± 0.09	0.85 ± 0.08	2.75 ± 0.11	6.86 ± 0.06
Huh7	0.44 ± 0.16	0.01 ± 0.01	0.44 ± 0.13	2.68 ± 0.11	1.16 ± 0.43
HL-60	0.19 ± 0.13	0.07 ± 0.01	0.72 ± 0.17	0.32 ± 0.15	1.14 ± 0.15

OF_MY_: *O. formosana* mycelium; CM_FB_: *C. militaris *fruiting body; CM_MY_: *C. militaris* mycelium; OS_FB_: *O. sinensis *fruiting body; OS_MY_: *O. sinensis *mycelium.
