# Supplementary material for: Evaluation of an Epitypified Ophiocordyceps formosana (Cordyceps s.l.) for Its Pharmacological Potential
Source: Evid Based Complement Alternat Med. 2015 Sep 15;2015:189891. doi: 10.1155/2015/189891 (PMC4587430; doi:10.1155/2015/189891)
Supplement: Supplementary file 1 — Degenerated primer sequences (Supporting Table 1) derived from the largest subunit of RNA polymerase II (RPB1), the second large subunit of RNA polymerase II (RPB2) and translation elongation factor-1α (EF1-α) genes were synthesized according to previous reports (30, 31, 32) and used for the molecular identifications of Cordyceps spp, (s. l.), including Ophiocordycpes formosana. The amplified 790-, 1062-, and 1068-base pair (bp) nucleotides, respectively, from our collected samples were sequenced and subjected to BLAST in the NCBI database (). The closest species and strains matched, NCBI accession number, and nucleotide identify of the 3 sequences were listed (Supporting Table 2). A list of nucleotide sequences of the 3 genes (i.e. RPB1, RPB2, and EF-1a) from 51 species/isolates was retrieved from the NCBI database following the criteria described by Sung et al or obtained from our sequenced PCR products of the O. formosana isolates (Supporting Table 3). [file 189891.f1.pdf]

Supporting table 1

Primer sequences used for the molecular identifications in this study

Target gene	Name of primers	Primer sequences	Reference
RPB1	cRPB1	CCWGGYTTYATCAAGAARGT	[25]
	RPB1cr	CCNGCDATNTCRTTRTCCATRTA	[25]
RPB2	fRPB2-5f	GAYGAYMGWGATCAYTTYGG	[26]
	fRPB2-7cr	CCCATRGCTTGYTTRCCCAT	[26]
EF1-α	EF1-2218R	ATGACACCRACRGCRACRGTYTG	[27]
	EF1-983F	GCYCCYGGHCAYCGTGAYTTYAT	[27]

Supporting table 2

Comparisons of the homologous strains of *O. formosana* via BLAST of the NCBI GenBank database based on the *RPB1*, *RPB2*, and *EF1-α* genes.

Genetic markers	Closest species and strains matched	Nucleotide identity	Accession number
RPB1	*Cordyceps formosana* TNM F13893	99%(635/635)	KJ878988.1
RPB2	*Cordyceps formosana* TNM F13893	100% (544/544)	KJ878943.1
EF1-α	*Cordyceps formosana* TNM F13893	99%(847/849)	KJ878956.1

Supporting table 3

Nucleotide sequences used in phylogenetic analysis and their accession numbers.

Species names	Accession numbers of genes		
	RPB1	*RPB2*	*EF1-α*
Ophiocordyceps irangiensis OSC 128579	EF469089	EF469107	EF469060
Ophiocordyceps rhizoidea NHJ 12529	EF468872	EF468922	EF468765
Ophiocordyceps rhizoidea NHJ 12522	EF468873	EF468923	EF468764
Ophiocordyceps sinensis EFCC 7287	EF468874	EF468924	EF468767
Cordyceps stylophora OSC 110999	EF468882	EF468931	EF468777
Ophiocordyceps entomorrhiza KEW 53484	EF468857	EF468911	EF468749
Elaphocordyceps subsessilis OSC 71235	EF469090	EF469108	EF469061
Ophiocordyceps gracilis EFCC 8572	EF468859	EF468912	EF468751
Ophiocordyceps gracilis EFCC 3101	EF468858	EF468913	EF468750
Ophiocordyceps heteropoda EFCC 10125	EF468860	EF468914	EF468752
Ophiocordyceps formosana TNM F13893	KJ878988	KJ878943	KJ878956
Ophiocordyceps formosana MUCHO 815	KR052521	KR052522	KR052523
Ophiocordyceps formosana NTU 00035	KT275190	KT275191	KT275192
Ophiocordyceps konnoana EFCC 7315	EF468861	EF468916	EF468753
Cordyceps ravenelii OSC 110995	DQ522379	DQ522430	DQ522334
Ophiocordyceps nigrella EFCC 9247	EF468866	EF468920	EF468758
Ophiocordyceps variabilis OSC 111003	EF468885	EF468933	EF468779
Cordyceps s variabilis ARSEF 5365	DQ522386	DQ522437	DQ522340
Haptocillium zeosporum CBS 335.80	EF469091	EF469109	EF469062
Paecilomyces lilacinus CBS 431.87	EF468897	EF468940	EF468791
Paecilomyces lilacinus CBS 284.36	EF468898	EF468941	EF468792
Nectria sp. CBS 478.75	EF469097	EF469115	EF469068
Torrubiella luteorostrata NHJ 12516	EF468905	EF468946	EF468800
Aschersonia placenta BCC 7869	EF469085	EF469104	EF469056
Shimizuomyces paradoxus EFCC 6279	EF469100	EF469117	EF469071
Shimizuomyces paradoxus EFCC 6564	EF469101	EF469118	EF469072
Claviceps purpurea SA cp11	EF469087	EF469105	EF469058
Balansia epichloe AEG 96-1 5a	EF468851	EF468908	EF468743
Pochonia bulbillosa CBS 145.70	EF468902	EF468943	EF468796
Tolypocladium parasiticum ARSEF 3436	EF468904	EF468945	EF468799
Pochonia rubescens CBS 464.88	EF468903	EF468944	EF468797
Pochonia chlamydosporia CBS 504.66	EF469098	EF469120	EF469069
Nomuraea rileyi CBS 806.71	EF468893	EF468937	EF468787
Paecilomyces carneus CBS 239.32	EF468894	EF468938	EF468789

Paecilomyces carneus CBS 399.59	EF468895	EF468939	EF468788
Paecilomyces marquandii CBS 182.27	EF468899	EF468942	EF468793
Cordyceps sp. OSC 110996	EF468880	EF468928	EF468773
Cordyceps sp. NHJ 12118	EF468878	EF468927	EF468768
Torrubiella wallacei CBS 101237	EF469102	EF469119	EF469073
Cordyceps cardinalis OSC 93610	EF469088	EF469106	EF469059
Lecanicillium attenuatum CBS 402.78	EF468888	EF468935	EF468782
Cordyceps militaris CM01	Whole genome blast	Whole genome blast	Whole genome blast
Cordyceps kyusyuensis EFCC 5886	EF468863	EF468917	EF468754
Microhilum oncoperae AFSEF 4358	EF468891	EF468936	EF468785
Cordyceps takaomontana NHJ 12623	EF468884	EF468932	EF468778
Cordyceps ochraceostromata ARSEF 5691	EF468867	EF468921	EF468759
Cordyceps bifusispora EFCC 8260	EF468855	EF468910	EF468747
Cordyceps bifusispora EFCC 5690	EF468854	EF468909	EF468746
Lecanicillium psalliotae CBS 532.81	EF469096	EF469112	EF469067
Lecanicillium psalliotae CBS 101270	EF469095	EF469113	EF469066
Torrubiella alba CBS 726.73a	EF468887	EF468934	EF468781
Roumegueriella rufula GJS 91-164	EF469099	EF469116	EF469070

Supporting text 1

Descriptions of *Ophiocordyceps formosana*

***Combinatio nova*:**

Ophiocordyceps formosana (Kobayasi & Shimizu) Wang, Tsai, Tzean & Shen *comb. nov.*

Mycobank: MB811035

≡ ***Cordyceps formosana*** Kobayasi & Shimizu, Bull. Natn. Sci. Mus.: 113 (1981)

Ascostroma arises from the head or abdomen of an insect host (darkling beetle larva, Tenebrionoidea). Stroma stalk is long-cylindrical, 10-30 mm × 0.5-2 mm, orange (6A7) in color, with short hairs, without membranous sheath. Stroma head is oblong, 4-6 mm × 1-4 mm, consists of epidermal pseudoparenchymatous tissue. Perithecia are embedded, ovoid in shape, brownish orange (7C8) in color, 360-480 × 240-320 µm, with ostioles 60 µm in width, perithecial wall 20 µm in thickness, covered with 40-µm-thick epidermal tissue; ascus is long-cylindrical, with attenuated base, 6.5-7.9 µm × 160-240 µm, apex is thick dome-shaped, with narrow slit, 3.9-5.3 × 3.2-4.6 µm; ascospores are 8 in number, hyaline, cylindrical and filamentous, usually fragmenting into many partspores, showing cylindrical and truncated, 2.6-3.0 × 6.5-7.3 µm. Colonies on potato dextrose agar (PDA) appear orange (5A7) to white, pulvinate to umbonate, mycelium white, velutinous to floccose, exudating orange to pink droplets.

Anamorphic state: the *Hirsutella*-type. The conidiogenous cell exhibits a monophialidic, hyaline, elongated-ampuliform, 1.5-2.3 × 8.6-17.0 µm. Conidia hyaline is cylindrical, 1.6-2.3 × 3.6-6.9 µm.

Habitats: growing on a darkling beetle larva (Tenebrionoidea) on fallen decayed logs.

Known distribution: Lalashan, Taoyuan county, Taiwan. Fonghuangshan, Nanto county, Taiwan. Guanwu, Hsinchu county, Taiwan. Huangshan, Anhui Providence, China.

Specimen examined: MUCHO 815, Collected and examined on August 15, 2013, from Lalashan, Taoyuan county, Taiwan, GPS altitude: 1600 m, latitude: 24° 42' 1.20" N, longitude: 121° 25' 49.20" E. collector, Y.-W. Wang, S.-H. Tsai, T.-W. Hong, J.-Y. Lai, T.-L. Shen.
